# Circulating Adiponectin and Risk of Endometrial Cancer

**DOI:** 10.1371/journal.pone.0129824

**Published:** 2015-06-01

**Authors:** Qiaoli Zheng, Haijian Wu, Jiang Cao

**Affiliations:** 1 Clinical Research Center, The Second Affiliated Hospital, School of Medicine, Zhejiang University, 88 Jiefang Road, Hangzhou, Zhejiang Province, China; 2 Department of Dermatology, Sir Run Run Shaw Hospital, School of Medicine, Zhejiang University, 3 Qingchun Road East, Hangzhou, Zhejiang Province, China; 3 Department of Neurosurgery, The Second Affiliated Hospital, School of Medicine, Zhejiang University, 88 Jiefang Road, Hangzhou, Zhejiang Province, China; Ehime University Graduate School of Medicine, JAPAN

## Abstract

**Background:**

Adiponectin is an insulin-sensitizing hormone produced by adipocytes. It has been suggested to be involved in endometrial tumorigenesis. Published data have shown inconsistent results for the association between circulating adiponectin levels and endometrial cancer. In this study, we conducted a meta-analysis to evaluate the predictive value of circulating adiponectin levels on the development of endometrial cancer.

**Methods:**

PubMed, Embase, ISI web of knowledge, and Cochrane databases were searched for all eligible studies, and the summary relative risk (SRR) was calculated. Additionally, we performed dose-response analysis with eight eligible studies.

**Results:**

A total of 1,955 cases and 3,458 controls from 12 studies were included. The SRR for the ‘highest’ vs ‘lowest’ adiponectin levels indicated high adiponectin level reduced the risk of endometrial cancer [SRR = 0.40, 95% confidence interval (CI), 0.33–0.66]. Results from the subgroup analyses were consistent with the overall analysis. The SRR for each 1 µg/ml increase of adiponectin indicated a 3% reduction in endometrial cancer risk (95% CI: 2%–4%), and a 14% reduction for each increase of 5 µg/ml (95% CI: 9%–19%). No evidence of publication bias was found.

**Conclusions:**

This meta-analysis demonstrates that low level of circulating adiponectin is a risk factor for endometrial cancer.

## Introduction

Endometrial cancer is one of the most frequently diagnosed diseases in gynecologic cancers, with an estimated 280,000 new cases per year worldwide [1]. Obesity increases the risk of endometrial cancer, with an overall risk ratio of 1.60 (p < 0.0001) per 5 kg/m^2^ increase in body mass index (BMI) showed by a large meta-analysis [2], which may be caused by insulin resistance, increased sex steroid, chronic systemic inflammation and alterations of the levels of adipocyte-derived factors [3,4].

Adipose tissue secretes a number of bioactive substances including adiponectin, leptin, resistin, and tumor necrosis factor-γ (TNF-γ) [5]. Adiponectin is the most abundant adipocyte-derived factor, which is an insulin-sensitizing hormone and suggested to be involved in endometrial tumorigenesis [6]. The anti-angiogenic, anti-inflammatory and anti-apoptotic properties of adiponectin may mediate its anti-tumor effects [6]. The circulating adiponectin levels were low in obesity and increased after severe weight loss [7,8]. Experimental evidence suggests that the relationship between adiponectin concentrations and BMI was inverse [9]. Although there were lines of *in vitro* and *ex vivo* evidence for a causal role of adiponectin in endometrial cancer [10], results from epidemiologic studies are inconsistent, and most studies are limited to small sample size. In some case-control studies, high circulating levels of adiponectin were shown to be associated with a significant decrease in risk of endometrial cancer [11–17], while other studies showed no significant associations [18–21]. As circulating adiponectin level is an easily monitored marker and may be useful in prevention and early diagnosis of endometrial cancer, it will be of great importance to clarify the association between adiponectin levels and endometrial cancer risk. In this work, we conducted a meta-analysis with all eligible studies to assess the association between circulating levels of adiponectin and endometrial cancer risk. We also performed a dose-response meta-analysis and examined the possibility of both the linear and nonlinear associations.

## Materials and Methods

### Literature search

PubMed, Embase, ISI web of knowledge, and Cochrane databases were searched with the following terms: “endometrial” or “endometrium” and “neoplasm” or “cancer” or “carcinoma” and “adiponectin” or “ACDC” or “ADPN” or “APM1” or “APM-1” or “GBP28” or “ACRP30” or “ADIPOQ”. Additional articles were identified from searching the bibliographies of retrieved articles.

### Study selection

All articles were independently reviewed by two investigators. Studies were included if they met the following criteria: 1) the study evaluates the relationship between circulating adiponectin levels and endometrial cancer risk; 2) the study is a case-control or cohort study; 3) the study reports relative risk estimates or crude data for circulating adiponectin levels; 4) the study which reports the estimates for at least three categories of adiponectin levels is included for dose-response analysis. Studies were excluded if they: 1) are reviews without original data; 2) reported overlapping data from the same study population; 3) did not compare a formally recognised study design (such as cohort studies or case-control studies); 4) were available only in abstract.

### Data extraction

Data were extracted by two independent investigators using a predefined database. The following data were included: the first author’s surname; country of origin; year of publication; study name and study period; study design; sample size; mean age of individuals; menopausal status of individuals; laboratory assays for adiponectin levels; relative risk estimate [RR, 95% confidence interval (CI)] for “highest” vs “lowest” category [22] of adiponectin or dose-response (DR), and adjustment factors.

### Statistical analysis

We combined both odds ratios (ORs) and relative risks (RRs), for calculating the summary relative risk (SRR). The multivariate-adjusted risk estimates and corresponding confidence intervals were transformed into log relative risks for meta-analyses [23]. Pooled estimates of SRR were calculated by using an inverse-variance weighted random-effects model for the ‘highest’ vs the ‘lowest’ category of baseline adiponectin concentration [24]. The *I* ^*2*^ test [25] and Cochran’s Q-statistic test [26] were used to assess between-study heterogeneity. *I* ^*2*^ values of 0–25%, 25–50% and 50–75% indicate no, low, and moderate heterogeneity, respectively. A low p-value of Cochran’s Q-statistic (<0.05) indicates significant heterogeneity among studies. To investigate the effect of potential confounders, we conducted subgroup analyses by considering all the possible factors, such as study population, menopausal status of individuals, and adjustment factors for age, BMI, hypertension and diabetes. To evaluate the stability of the pooled estimates, we performed sensitivity analysis by examining changes in results after sequential omission of individual studies.

The summary estimate of the dose-response effect of adiponectin levels on endometrial cancer risk was calculated using fixed-effects model. Generalized least-squares regression was utilized to estimate the linear trend in SRR, while the method of restricted cubic spline was applied for nonlinear trend estimation [27]. Publication bias was graphically assessed using the Begg and Egger’s test [28,29]. All of the above analyses were performed by using STATA version 12 (StataCorp, College Station, TX).

## Results

### Study characteristics

**Fig 1:**
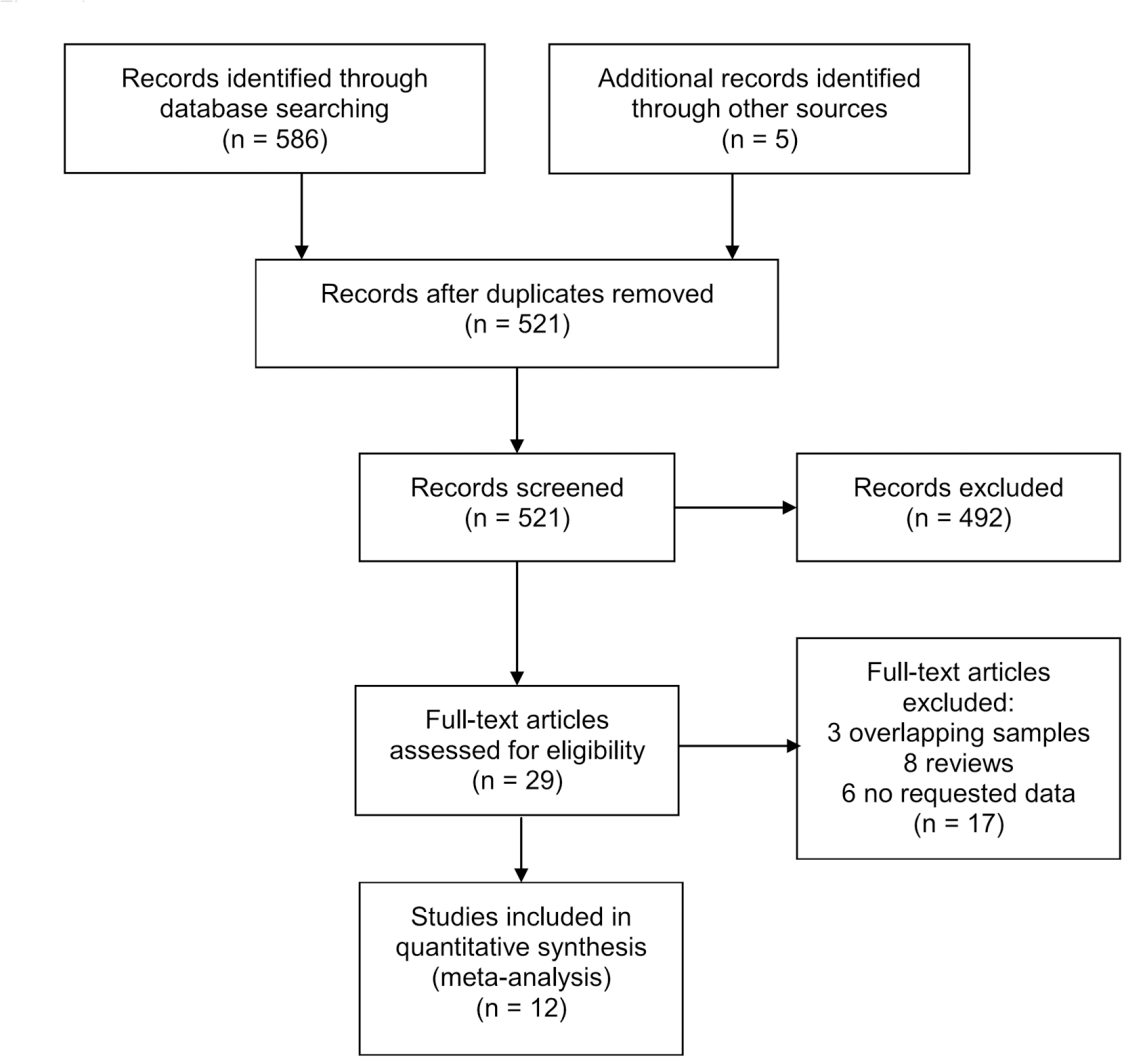
Flowchart of the study selection process.

A total of 12 eligible studies [11–21,30] including 1,955 cases and 3,458 controls were identified through search process (Fig 1). Among them, one study reported results from dose-response analyses [30], while the other 11 studies reported ORs for several categories of circulating adiponectin level [11–21]. The main characteristics of these studies are presented in Table 1. Five studies were conducted in North-America [12,14,16,19,20], four in Europe [11,13,15,30], and three in Asia [17,18,21]. Of the 12 case-control studies, five were hospital-based [11,15,17,18,30], three were population-based [12,14,21], and four were nested studies [13,16,19,20]. Adiponectin levels were measured by enzyme-linked immunosorbent assay (ELISA) in nine studies [12–15,17–21] and by radioimmunoassay (RIA) in three studies [11,16,30]. In one article the authors calculated RR [13], the other studies reported ORs [11,12,14–21,30]. A total of eight studies with sufficient data were included in dose-response meta-analysis [11,13–16,18–20].

**Table 1:** Characteristics of studies included in the meta-analysis.

Author, year, country	Study name, study period	Study design	No. cases/controls	Mean age (years) cases/controls	Menopausal status	Assay method	Adiponectin levels (µg/ml) cases/controls	RR (95% CI) for “highest” vs “lowest” category of adiponectin or dose-response (DR)	Adjustment factors
Petridou *et al*.,2003, Greece	1999	Case-control (HB)	84/84	NA	Mainly post	RIA	NA/13.53	0.80 (0.58–1.10) (DR)	Age, education, height, BMI before onset of symptoms, Age at menarche, Ever pregnant
Maso *et al*., 2004, Italy	1991–2002	Case-control (HB)	87/132	62/61	51(23%) pre, 168(77%) post	RIA	11.4/16.0	0.30 (0.14–0.68)	Age, education, parity, BMI, smoking, HRT
Soliman *et al*., 2006, USA	2000–2004	Case-control (PB)	117/238	66.6/61.2	NA	ELISA	8.88/14.82	0.10 (0.04–0.24)	Age, BMI, diabetes, hypertension
Cust *et al*., 2007, Europe	EPIC, 1999–2003	Case-control (Nested)	284/548	56.9/56.9	159(19%) pre, 563(68%) post, 110(13%) NA	ELISA	8.4/9.9	0.56 (0.36–0.86)	BMI
Ashizawa *et al*., 2010, Japan	2004–2008	Case-control (HB)	150/150	59.5/57.5	All post	ELISA	6.2/9.0	0.6 (0.3–1.2)	Age, BMI, diabetes mellitus, hypertension
Soliman *et al*., 2011, USA	NHS, 1989–2004	Case-control (Nested)	146/377	57/57	87(17%) pre, 436(83%) post	ELISA	12.9/12.9	0.98 (0.57–1.68)	BMI, parity, diabetes
Friedenreich *et al*., 2012, Canada	2002–2006	Case-control (PB)	549/1036	59/59	163(10%) pre, 1422(90%) post	ELISA	11.6/14.6	0.47 (0.33–0.69)	Age at reference, nulliparous (vs multiparous), HRT, menopausal hormone use, hypertension, weight at reference, date, and waist-to-hip ratio
Dallal *et al*., 2013, USA	FIT, 1992–2004	Case-control (Nested)	62/124	67.4/67.5	All post	ELISA	14.3/14.6	1.31 (0.55–3.12)	BMI
Erdogan *et al*., 2013, Turkey	2012–2013	Case-control (HB)	60/70	56.6/49.7	All post	ELISA	4.09/17.13	0.09 (0.02–0.36)	Age, BMI, HOMA-TR, QUICK-I
Luhn *et al*., 2013, USA	PLCO, 1993–2001	Case-control (Nested)	167/327	66.4/NA	All post	RIA	12.16/14.77	0.48 (0.29–0.80)	family history of breast or endometrial cancer, education level, parity, history of diabetes diagnosis, oral contraceptive use, and current smoking status
Ma *et al*., 2013, China	2008–2010	Case-control (HB)	206/310	53.2/53.3	NA	ELISA	2.33/2.58	0.52 (0.32–0.83)	BMI, glucose, cholesterol, triglycerides, high density lipoprotein cholesterol, age, insulin and leptin-to-adiponectin (L/A)
Ohbuchi *et al*., 2013, Japan	2006–2007	Case-control (PB)	43/62	61.2/58.1	NA	ELISA	4.9/7.0	0.50 (0.07–3.45)	Age, BMI, hypertension, and diabetes mellitus

RR, relative risk; CI, confidence interval; DR, dose-response; HB, hospital-based; NA, not applicable; RIA, radioimmunoassay; BMI, body mass index; pre, premenopause; post, postmenopause; HRT, hormone replacement therapy; PB, population-based; ELISA, enzyme-linked immunosorbent assay; EPIC, European Prospective Investigation into Cancer and Nutrition cohort study; NHS, Nurses’ Health Study; FIT, the Fracture Intervention Trial; HOMA-IR, the homeostasis model assessment of insulin resistance; QUICKI, quantitative insulin sensitivity check index; PLCO, Prostate, Lung, Colorectal and Ovarian cancer screening trial.

### “Highest” vs “lowest” category of adiponectin

**Fig 2:**
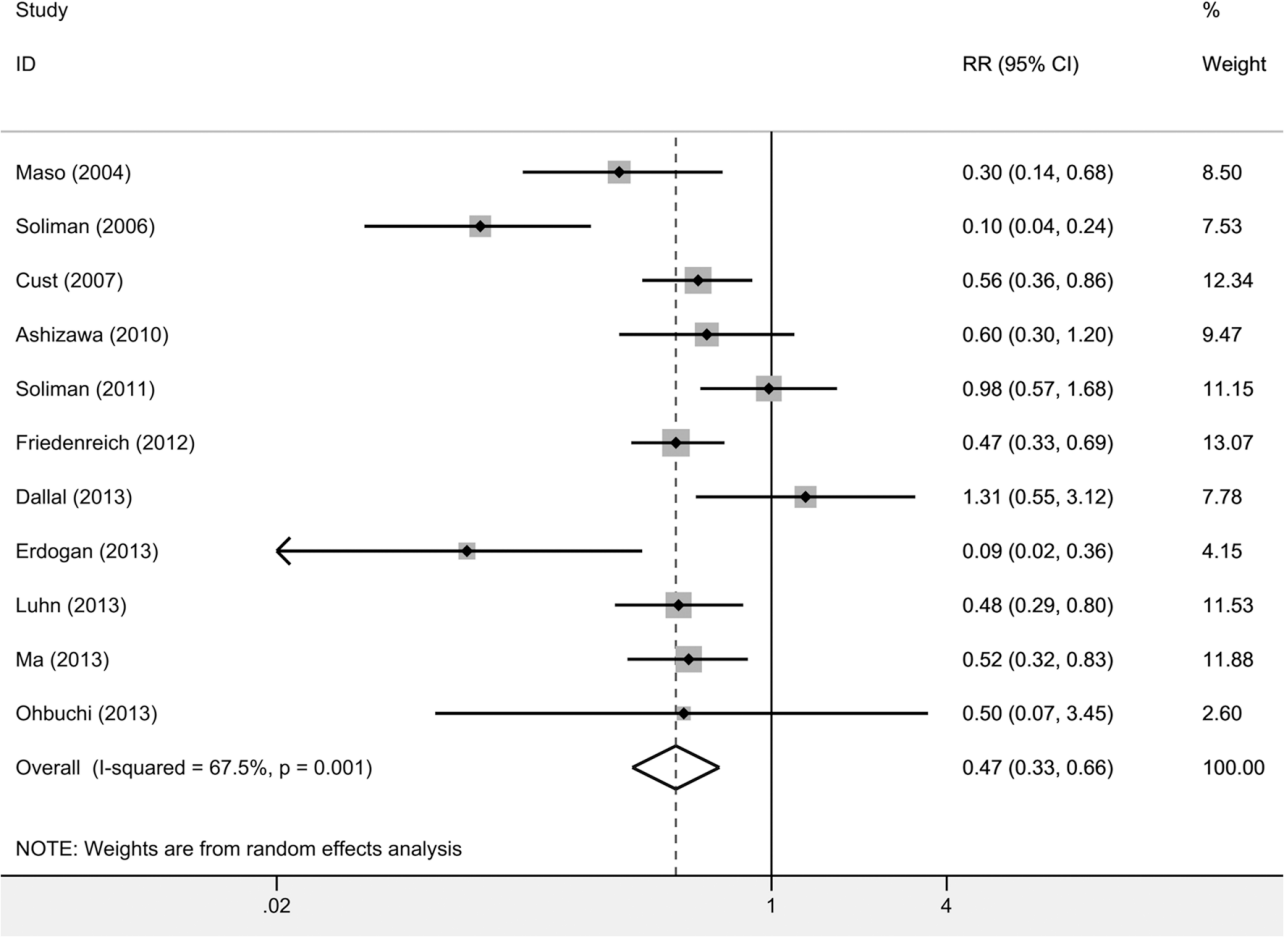
Forest plot of the ‘highest’ vs the ‘lowest’ category of circulating adiponectin and endometrial cancer risk. RR, relative risk; CI, confidence interval.

The SRR for the “highest” vs “lowest” category of adiponectin levels was 0.47 (95% CI: 0.33–0.66, p < 0.001), which indicated a 53% reduction in endometrial cancer risk (Fig 2) [11–21,30]. Some evidence of between-group heterogeneity was found in the analysis (*I* ^*2*^ = 67.5%, p = 0.001). Therefore, we conducted subgroup analysis to evaluate the influence of single factor that might have impact on the result. Table 2 demonstrates the results from subgroup analyses. In the subgroup analyses by districts and several adjustment factors, including BMI, hypertension and diabetes, the results were basically consistent with overall result. However, the “lowest” category of adiponectin didn’t significantly increase the risk of endometrial cancer in the premenopausal women (p = 0.807), neither in the group not adjusted for age (p = 0.099). Compared with the overall analysis, lower evidence of heterogeneity was found in postmenopausal women (*I* ^*2*^ = 53.8%) [11,13,15,16,18–20], in studies adjusted for age (*I* ^*2*^ = 64.8%) [11,12,14,15,17,18,21], and in studies not adjusted for age (*I* ^*2*^ = 53.9%) [13,16,19,20], hypertension (*I* ^*2*^ = 64.2%) [11,13,15–17,19,20] and diabetes (*I* ^*2*^ = 58.8%) [11,13–15,17,20]. No evidence of substantial heterogeneity was found in premenopausal women (*I* ^*2*^ = 0.0%) [11,13,19], in studies conducted in Asia (*I* ^*2*^ = 29.1%) [17,18,21], and in studies not adjusted for BMI (*I* ^*2*^ = 0.0%) [14,16].

**Table 2:** Subgroup analyses for circulating adiponectin and endometrial cancer risk.

	No. of studies	RR (95% CI)	P(Z)	*I* ^*2*^	P(Q)	Reference
Overall	11	0.47 (0.33–0.66)	<0.001	67.5%	0.001	[11–21,30]
District						
Asia	3	0.54 (0.37–0.80)	0.002	0.0%	0.943	[17,18,21]
Europe	3	0.31 (0.13–0.73)	0.007	70.2%	0.035	[11,13,15]
North-America	5	0.51 (0.27–0.95)	0.034	82.6%	<0.001	[12,14,16,19,20]
Menopausal status						
Pre	3	0.92 (0.45–1.87)	0.807	29.1%	0.244	[11,13,19]
Post	7	0.50 (0.33–0.76)	0.001	53.8%	0.043	[11,13,15,16,18–20]
Combined	4	0.34 (0.18–0.66)	0.001	72.8%	0.012	[12,14,17,21]
Adjustment factors						
Age						
Yes	7	0.34 (0.21–0.54)	<0.001	64.8%	0.009	[11,12,14,15,17,18,21]
No	4	0.71 (0.47–1.07)	0.099	53.9%	0.089	[13,16,19,20]
BMI						
Yes	9	0.45 (0.28–0.73)	0.001	73.7%	<0.001	[11–13,15,17–21]
No	2	0.47 (0.35–0.64)	<0.001	0.0%	0.948	[14,16]
hypertension						
Yes	5	0.35 (0.16–0.74)	0.006	73.3%	0.010	[12,14,18,21]
No	6	0.54 (0.36–0.81)	0.002	64.2%	0.010	[11,13,15–17,19,20]
diabetes						
Yes	5	0.44 (0.22–0.91)	0.027	78.4%	0.001	[12,16,18,19]
No	6	0.48 (0.33–0.71)	<0.001	58.8%	0.033	[11,13–15,17,20]

RR, relative risk; CI, confidence interval; pre, premenopause; post, postmenopause; BMI, body mass index.

### Dose-response meta-analysis

We evaluated the dose-response effect of adiponectin levels on endometrial cancer risk. An inverse linear dose-response correlation was observed from pooled 8 studies (p < 0.001), with a 3% (95% CI: 2%-4%) reduction of endometrial cancer risk for each 1 g/ml increase of adiponectin, and a 14% (95% CI: 9%-19%) reduction for each 5 g/ml increase of adiponectin. Additionally, a statistically nonlinear correlation between adiponectin level and endometrial risk was also observed (p < 0.001). No evidence of heterogeneity was observed when we calculated either the linearity (p = 0.10) or the nonlinearity (p = 0.25).

### Sensitivity analysis and publication bias

We conducted a sensitivity analysis in which individual studies were sequentially omitted and the rest analyzed. The results indicated none of the single studies significantly affected the SRRs. We did not detect any publication bias by the Egger’s (p = 0.497) or Begg’s test (p = 0.533).

## Discussion

Our meta-analysis suggests an inverse correlation between circulating adiponectin level and endometrial cancer, with a 53% reduction in risk for higher levels. The result was consistently significant in all sensitivity analyses and in all populations studied. However, no significant associations were observed in premenopausal women, and in studies not adjusted for age. The dose-response analysis shows that each 1 g/ml increase of adiponectin corresponds to 3% reduction in risk of endometrial cancer. The overall dose-response association was estimated by Petridou *et al*., but it was not significant which might due to the limited number of subjects analyzed [30]. Moreover, we found a statistically nonlinear correlation between adiponectin and endometrial cancer risk.

In our stratified analysis by menopausal status, we only found a statistically significant association between adiponectin level and endometrial cancer risk in postmenopausal women. Indeed, three studies including only 297 subjects reported nonsignificant results among premenopausal women [11,13,19]. The small sample size may not be sufficiently powerful to detect a difference between the groups. On the other hand, endometrial cancer is more common in postmenopausal women than in premenopausal women [31]. Obesity is a strong risk factor for endometrial cancer with the level of risk related to BMI [32]. When considering the BMI, we found that lower levels of circulating adiponectin increased endometrial cancer risk in studies adjusted for BMI, which was consistent with the overall results. We observed a statistically significant heterogeneity across the studies included in the meta-analysis. Some evidence of heterogeneity was also observed in subgroup analyses, therefore we calculated the summary estimates using the random effect models which is more conservative.

Many studies have addressed on the mechanisms for the role of adiponectin on reducing endometrial cancer risk. One major mechanism is that adiponectin can decrease blood insulin levels [5,33–35], and therefore can inhibit cancer development, as the insulin-upregulated estrogen is one of the predominant risk factor for endometrial cancer [13,20,32,36,37]. In addition, the association between adiponectin level and endometrial cancer suggests that insulin resistance may play an important role in endometrial carcinogenesis [11,12,14,16–19,21]. Dal Maso *et al*. also observed that high-glycemic load diets, which cause high levels of blood glucose and insulin, are directly related to endometrial cancer risk [11]. Inflammation is considered to play an important role in the initiation and progression of tumor [38]. Adiponectin may have strong anti-inflammatory activity, and could also potentially reduce the risk of endometrial cancer thereby [34,39–44]. Furthermore, adiponectin-induced caspase-mediated endothelial cell apoptosis and showed anti-angiogenesis and anti-tumor activity [45]. Moreover, adiponectin may inhibit estrogen receptor α and vascular endothelium growth factor, thus suppressing cell proliferation, invasiveness and angiogenesis *in vitro* [17]. Finally, adiponectin can suppress the proliferative actions of several mitogenic growth factors by blocking their interaction with the membrane receptors [44,46]. It has been assumed that adiponectin may prevent carcinogenesis through invoking AMPK and suppressing phosphatidylinositol 3-kinase (PI3K)/AKT/mTOR signaling [15]. Therefore the circulating adiponectin plays a protective role in the development of endometrial cancer [11–17], which is accordance with the conclusion of our meta-analysis.

In addition, adiponectin circulates in plasma in three forms of low-molecular-weight (LMW) oligomeric complex of a trimer, a middle-molecular-weight (MMW) complex of two trimers and a high-molecular-weight (HMW) complex of up to six trimers [47]. Experimental evidence suggests that different forms of adiponectin may have distinct biological effects, such that the HMW form was suggested to be more closely related to insulin sensitivity, while complexes with lower molecular weight may have stronger anti-inflammatory potential [48]. A study reported that low serum level of MMW adiponectin was the only independent risk factor for endometrial cancer [21]. Further studies are needed to identify the correlation of serum levels or the ratios of different adiponectin isoforms with the risk of endometrial cancer.

The current meta-analysis supports the direct link between lower level of circulating adiponectin and increased endometrial cancer risk. By combining information from all relevant studies, our meta-analysis overcomes the limitations of time period and the specific subject population of study, increases the sample size and thus the power to study effects of interest. Although endometrial cancer is a frequent gynecologic cancer, it is a relatively low-incidence disease compared to other common cancers such as breast cancer, lung cancer and colorectal cancer, odds ratio (OR) can be used as a good estimate of relative risk. Therefore it is appropriate to combine both ORs and RRs for calculating a summary relative risk. However, this study still has several limitations. First, selection bias may exist, because parts of control subjects are hospital-based, who might have benign disease and different risks for developing endometrial cancer. Second, adjustments for the clinicopathological parameters, which may result in measurement error, together with other inter-study variances enhance the between-study heterogeneity. Third, some of the findings in subgroups are limited by small sample sizes. Further large prospective cohort studies should be carried out to investigate the optimal level of adiponectin with a protective effect on endometrial cancer. Although various pharmacological and non-pharmacological interventions have been adopted to influence adiponectin levels, the clinical relevance of the elevated adiponectin level to the reduction of the endometrial cancer risk remains to be determined [49].

Though adiponectin is expressed predominantly by adipose tissue, its plasma concentrations have been demonstrated to be negatively correlated with BMI, insulin resistance and insulin concentrations [50]. The expression of adiponectin is modulated at both genomic and epigenomic levels [51,52], therefore it is important to investigate the mechanisms for the down-regulation of adiponectin in endometrial cancer patients in the future for better prevention and treatment. In summary, this meta-analysis supports that lower level of circulating adiponectin is a risk factor for endometrial cancer. As a meta-analysis includes more cases than an individual study, it may draw a more convincing conclusion; in this case, the result of the study by Dallal CM *et al*. which showed that RR is in the opposite direction as we hypothesized (Fig 2) may result from the smaller sample size [20]. The result of this meta-analysis is strengthened by biologically plausible mechanisms underlying the roles adiponectin plays in cancers. Adiponectin may serve as a risk biomarker for endometrial cancer, and may have important clinical relevance in the prevention of endometrial cancer.

## Supporting Information

S1 Checklist PRISMA checklist for this meta-analysis. (DOC)
